# 1103. Minocycline (MIN) Pharmacodynamics (PD) against *Stenotrophomonas maltophilia* (STM) in a Neutropenic Murine Thigh Infection Model

**DOI:** 10.1093/ofid/ofab466.1297

**Published:** 2021-12-04

**Authors:** Andrew J Fratoni, David P Nicolau, Joseph L Kuti

**Affiliations:** 1 Hartford Hospital, Hartford, Connecticut; 2 Center for Anti-Infective Research and Development, Hartford Hospital, Hartford, Connecticut

## Abstract

**Background:**

Antibiotic treatment options for serious STM infections are limited. MIN displays in vitro activity against STM; however, limited data supports optimal dosing for STM. Herein, we employed the murine neutropenic thigh infection model to assess MIN PD against STM.

**Methods:**

Four clinical STM isolates with MIN MICs 0.25 – 1 mg/L were included. Both thighs of neutropenic ICR mice were inoculated with bacterial suspensions of 10^7^ colony forming units (CFU)/mL. Mice received uranyl nitrate on Day -3 to provide predictable renal impairment. Two hours after inoculation, MIN or control was administered subcutaneously. Pharmacokinetic (PK) studies of 2.5, 25, 50, and 100 mg/kg were conducted. Previously reported protein binding of 78.1% was used to define free exposure. Dose ranging studies were conducted on all STM to assess in vivo activity over a range of MIN exposures. MIN total daily doses (TDD) of 10, 20, and 50 mg/kg were fractionated q24h, q12h, and q6h against a single STM to determine the PD index best correlated with reductions in CFU/mL. Efficacy was measured in log_10_CFU/thigh at 24h compared with 0h controls. Composite CFU data were fitted to an E_max_ model to determine the *f*AUC/MIC exposure for stasis and 1 log_10_ reduction.

**Results:**

MIN PK was linear up to 50 mg/kg and well described by a 1 compartment model with first order absorption and elimination. Mean PK parameters across the linear range were: Vd, 2.97 L/kg; K01, 10.62 1/h; and K10, 0.35 1/h. Mean ± SD bacterial burden at 0h across all isolates was 6.17±0.20 log_10_CFU/thigh. In 24h controls, bacterial growth was 7.90±0.85 log_10_CFU/thigh. A dose response was observed across all isolates using TDD of 2-300 mg/kg. PD indices correlated with CFU reductions as follows: *f*AUC/MIC (R^2^=0.613), *f*Cmax/MIC (R^2^=0.590), and %*f*T >MIC (R^2^=0.504). The *f*AUC/MIC needed for stasis and 1 log_10_ reduction at 24h was 9.6 and 23.6, respectively.

**Conclusion:**

These are the first data describing MIN PD against STM. Against these STM, MIN *f*AUC/MIC was the PD index best correlated with CFU reductions. The exposure thresholds defined in this study will be useful in designing optimal MIN dosing regimens for treating STM infections and re-assessment of the current susceptibility breakpoint.

The study was funded under FDA Contract 75F40120C00171.

**Disclosures:**

**David P. Nicolau, PharmD**, **Abbvie, Cepheid, Merck, Paratek, Pfizer, Wockhardt, Shionogi, Tetraphase** (Other Financial or Material Support, I have been a consultant, speakers bureau member, or have received research funding from the above listed companies.) **Joseph L. Kuti, PharmD**, **Allergan** (Speaker’s Bureau)**BioMérieux** (Consultant, Research Grant or Support, Speaker’s Bureau)**Contrafect** (Scientific Research Study Investigator)**GSK** (Consultant)**Merck** (Research Grant or Support)**Paratek** (Speaker’s Bureau)**Roche Diagnostics** (Research Grant or Support)**Shionogi** (Research Grant or Support)**Summit** (Scientific Research Study Investigator)

